# Longitudinal In Vivo Imaging at Single-Lesion Resolution Identifies Allele-Associated Response and Resistance Dynamics in EGFR-Mutant Lung Cancer

**DOI:** 10.3390/ijms27177727

**Published:** 2026-08-28

**Authors:** Eva Cabrera San Millan, Daniele Panetta, Paolo Armanetti, Mauro Quaglierini, Alessandro Zega, Raffaella Mercatelli, Emilia Bramanti, Luca Menichetti, Giorgia Maroni, Elena Levantini

**Affiliations:** 1 CNR-Consiglio Nazionale delle Ricerche, Istituto di Tecnologie Biomediche (ITB-CNR), Area della Ricerca di Pisa, 56124 Pisa, Italy; eva.cabrera@phd.unipi.it (E.C.S.M.); raffaella.mercatelli@cnr.it (R.M.); 2 CNR-Consiglio Nazionale delle Ricerche, Istituto di Fisiologia Clinica (IFC-CNR), Area della Ricerca di Pisa, 56124 Pisa, Italy; daniele.panetta@cnr.it (D.P.); paolo.armanetti@ifc.cnr.it (P.A.); mauro.quaglierini@cnr.it (M.Q.); alessandro.zega@cnr.it (A.Z.); luca.menichetti@cnr.it (L.M.); 3 CNR-Consiglio Nazionale delle Ricerche, Istituto di Chimica dei Composti OrganoMetallici (ICCOM-CNR), SS di Pisa, Area della Ricerca di Pisa, 56124 Pisa, Italy; emilia.bramanti@cnr.it; 4 Fondazione Pisana per la Scienza ONLUS (FPS), San Giuliano Terme, 56017 Pisa, Italy

**Keywords:** NSCLC, GEMM, EGFR, therapeutic resistance, micro-CT, 3D imaging

## Abstract

Acquired resistance to targeted therapies is inevitable in EGFR-mutant non-small cell lung cancer (NSCLC), yet the principles governing its emergence in vivo remain incompletely understood. In particular, how lesion-level response patterns vary across distinct EGFR allele contexts during therapy has not been systematically examined at single-lesion resolution. Here, we establish a longitudinal in vivo imaging platform enabling single-lesion resolution tracking of tumor behavior during therapy in genetically engineered mouse models representing clinically relevant EGFR alleles. Using high-resolution micro-computed tomography (micro-CT) and three-dimensional reconstruction, we monitor tumor growth, therapeutic response, and resistance during osimertinib treatment. EGFR genotype is associated with distinct patterns of tumor growth, response kinetics, and resistance timing. Therapeutic response is spatially heterogeneous, with coexisting lesions undergoing complete regression, persistence, or progression within the same lung. During treatment, spatially distinct lesion-level behaviors included persistent growth during therapy and initial regression followed by regrowth. These findings demonstrate the utility of longitudinal micro-CT imaging to investigate allele-associated differences in treatment response and resistance timing at single-lesion resolution in vivo.

## 1. Introduction

Non-small cell lung cancer (NSCLC) accounts for ~85% of lung cancer cases and remains the leading cause of cancer-related mortality [1,2]. Lung adenocarcinoma (LUAD), the most common subtype, is frequently driven by activating mutations in the epidermal growth factor receptor (EGFR), occurring in ~15–20% of Caucasian patients and associated with poor long-term clinical outcomes due to the inevitable emergence of therapeutic resistance [3,4,5].

The most prevalent EGFR alterations are in-frame deletions in exon 19 (Del19) and the L858R missense mutation in exon 21, which together account for ~85% of common EGFR activating mutations [4,6,7]. Patients harboring these mutations are initially sensitive to tyrosine kinase inhibitors (TKIs), which represent the standard first-line therapy [4,7]. Among them, osimertinib, a third-generation TKI, significantly improves progression-free survival (PFS) and overall survival (OS) compared with earlier-generation inhibitors [7,8], as it targets both activating EGFR mutations and T790M-mediated resistance arising during prior TKI therapy. However, despite initial responses, acquired resistance to osimertinib inevitably emerges.

Resistance to EGFR-targeted therapies is highly heterogeneous and arises through multiple mechanisms, including secondary EGFR alterations and activation of bypass signaling pathways [4,9,10,11,12,13]. Importantly, resistance is not a uniform process across tumors or patients, but rather reflects complex biological processes influenced by both genetic and non-genetic factors [14,15]. Clinical evidence further suggests that resistance patterns differ across EGFR genotypes, with variation in progression kinetics, secondary alterations, and clinical outcomes, supporting an allele-associated component of therapeutic escape [16,17,18,19,20,21]. Consistent with current models of therapeutic resistance, resistance is thought to arise through the selection of pre-existing and/or therapy-emergent resistant populations [22].

In parallel, increasing evidence supports the existence of drug-tolerant persister (DTP) cells, which survive initial therapy through reversible adaptive programs [22,23,24], and constitute a reservoir from which resistant disease may eventually emerge. Moreover, spatial intratumor heterogeneity contributes to variable tumor composition and has been demonstrated by multiregion sequencing studies in solid tumors [25]. Such heterogeneity gives rise to regionally distinct subclones with differential drug sensitivity within the same lesion [25,26,27]. Consistently, patients treated with EGFR-targeted therapies frequently exhibit mixed patterns of response and progression across lesions, reflecting underlying intrapatient heterogeneity and lesion-specific response patterns during therapy [28,29,30,31].

However, the determinants of allele-specific resistance remain incompletely understood, and lesion-level response patterns during therapy are not fully characterized at single-lesion resolution, limiting our ability to anticipate and intercept resistance progression [4,32,33,34,35,36].

Therapeutic response is not uniform across EGFR genotypes. Exon 19 deletions are generally associated with higher objective response rates (ORR) and longer progression-free survival (PFS) compared with L858R mutations, both in first-line and across broader clinical cohorts [37,38] reflecting biological and clinical heterogeneity among EGFR mutation subtypes [39]. Subgroup analyses further indicate that specific Del19 variants, particularly those initiating at E746, are associated with improved outcomes compared with other deletion patterns [40], highlighting clinically relevant diversity within EGFR exon 19 deletions. In T790M-positive cohorts, osimertinib also demonstrates improved efficacy in Del19 compared with L858R tumors [16].

To dissect these allele-specific effects, we employed genetically engineered mouse models (GEMMs) representing three clinically relevant EGFR driver alleles: exon 19 deletions (EGFR^D19^), L858R (EGFR^LR^), and L858R/T790M (EGFR^LT^). Mice were treated with osimertinib until resistance developed, generating osimertinib-resistant (OsiR) tumors. Using longitudinal high-resolution micro-computed tomography (micro-CT) and three-dimensional (3D) reconstruction, this in vivo platform enables lesion-level tracking of tumor growth, therapeutic response, and resistance timing across distinct EGFR genotypes while preserving the spatial distribution of lesions throughout therapy.

## 2. Results

### 2.1. Generation and Longitudinal Monitoring of Osimertinib-Resistant EGFR GEMMs Reveals Allele-Associated Differences in Tumor Growth and Resistance Timing

**Figure 1:**
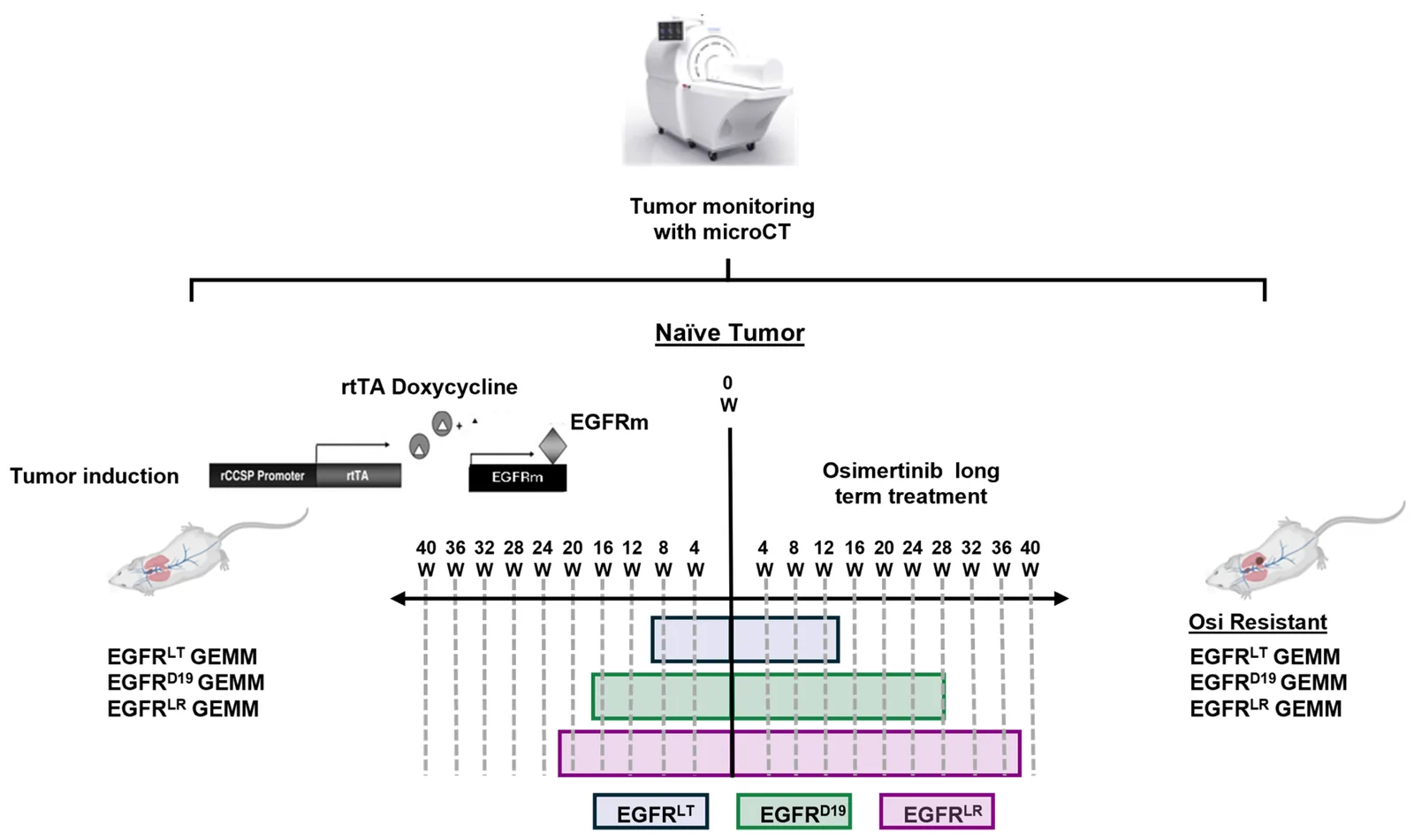
Experimental strategy for the generation of osimertinib-resistant EGFR GEMMs. Schematic overview of tumor induction and treatment timeline in EGFR-driven GEMMs. Tumors were induced by doxycycline-dependent expression of mutant EGFR alleles, followed by longitudinal monitoring with high-resolution micro-CT. W denotes weeks. After baseline imaging of naïve tumors (week 0), mice undergo continuous osimertinib treatment, leading to the emergence of osimertinib-resistant tumors. The timeline illustrates the duration of treatment and the micro-CT imaging schedule for the EGFR^LT^, EGFR^D19^, and EGFR^LR^ models.

To investigate the dynamics of osimertinib resistance in vivo, we generated OsiR GEMMs across three distinct EGFR mutant backgrounds (EGFR^D19^, EGFR^LR^, and EGFR^LT^) (Figure 1), inducing lung tumors via doxycycline-dependent activation of mutant EGFR and monitoring tumor growth longitudinally by high-resolution micro-CT.

Tumor initiation exhibited marked allele-specific differences: EGFR^LT^ tumors became detectable within 2–3 months of induction, EGFR^D19^ tumors after 4–5 months, and EGFR^LR^ tumors showed the longest latency, reaching comparable volumes only after 5–6 months. These findings indicate that different oncogenic EGFR alleles are associated with distinct tumor initiation kinetics in vivo prior to therapeutic intervention.

Once tumors reached measurable size, mice were treated with osimertinib to induce acquired resistance. The timing of resistance emergence, defined as tumor re-growth during continuous therapy, varied substantially among genotypes (Figure 1). EGFR^LT^ tumors developed resistance after ~3.5 months, EGFR^D19^ tumors after ~7 months, and EGFR^LR^ tumors after ~9.5 months of therapy. Notably, within this cohort, EGFR^LT^ tumors exhibited both the shortest tumor latency and the earliest resistance emergence, whereas EGFR^D19^ and EGFR^LR^ tumors displayed delayed tumor formation and prolonged sensitivity to osimertinib (OsiS).

Together, longitudinal micro-CT imaging revealed allele-associated differences in tumor onset, growth kinetics, and resistance timing across EGFR mutant genotypes in vivo.

### 2.2. Longitudinal Response Dynamics in EGFR^D19^ and EGFR^LR^ Tumors

Longitudinal micro-CT imaging was used to monitor tumor dynamics during osimertinib treatment. Tumor volumes were calculated relative to the pre-treatment (naïve) state, and 3D reconstructions were generated at defined stages to visualize lesion distribution and dynamics.

**Figure 2:**
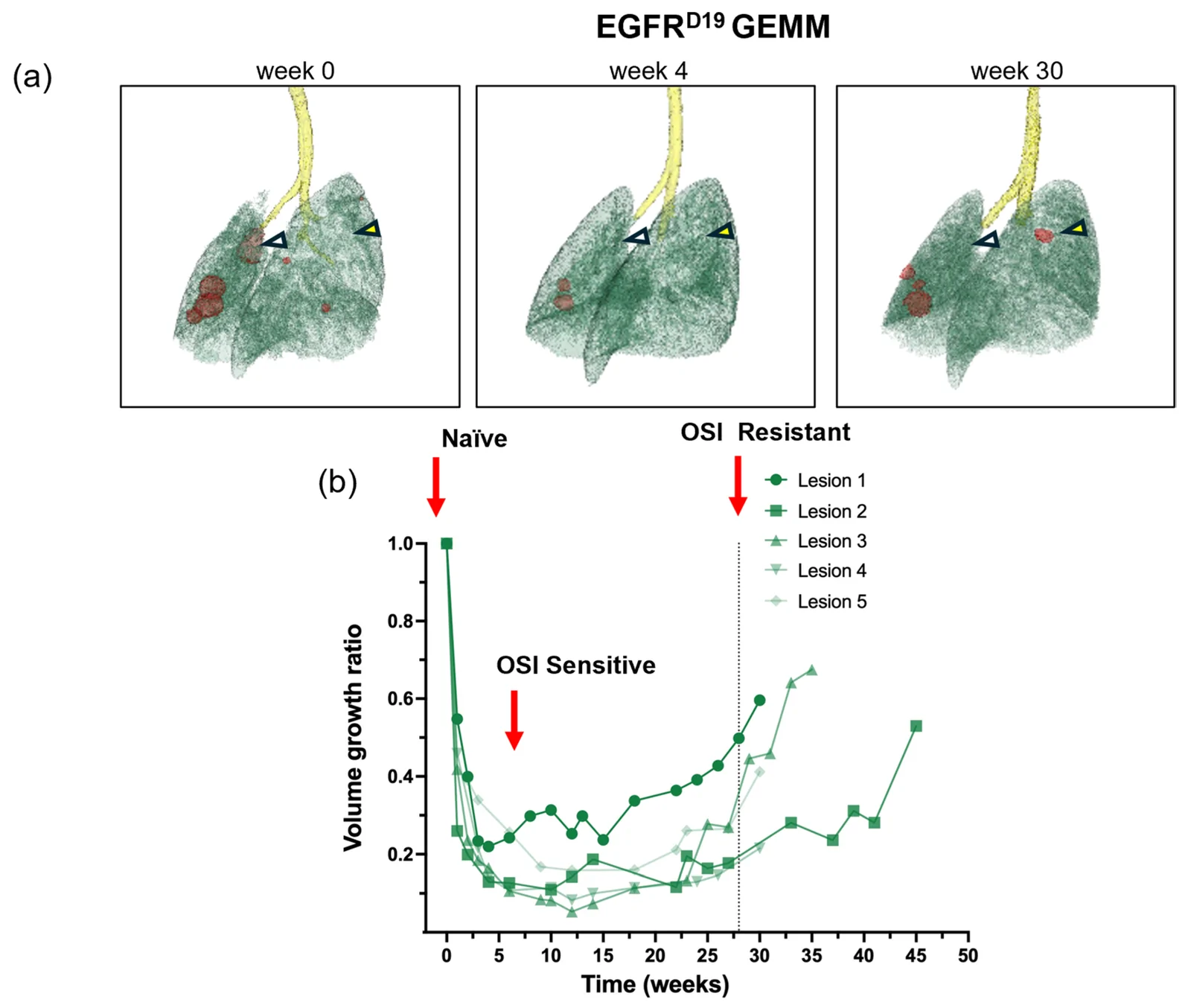
Generation of osimertinib-resistant EGFR^D19^ GEMMs. (**a**) Representative micro-CT-based 3D reconstructions showing lung tissue (green), tumor lesions (red), and trachea (yellow) at baseline (week 0, naïve stage), during response to osimertinib (week 4), and after acquisition of resistance at the end of treatment (week 30). White arrows indicate lesions that regress completely and do not re-grow, whereas yellow arrows mark lesions that become detectable during the resistant phase. (**b**) Tumor volume growth kinetics of individual lesions showing initial regression followed by tumor re-growth upon acquired resistance. Each curve indicates a representative lesion per mouse (*n* = 3 mice; *n* = 5 lesions) that remained measurable throughout treatment. Red arrows indicate naïve, responding (OsiS), and resistant-stage (OsiR) time points used for longitudinal lesion tracking.

**Figure 3:**
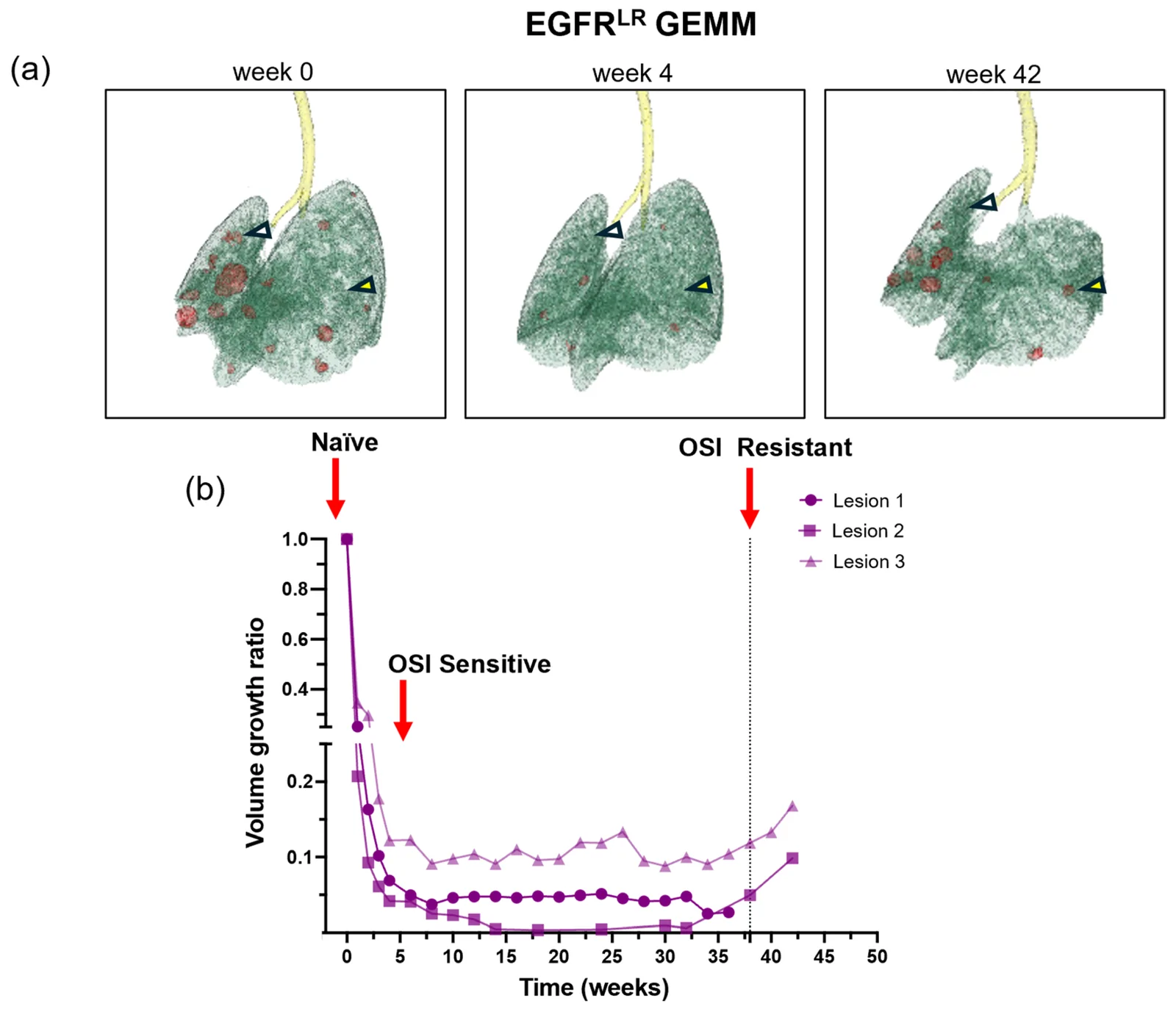
Generation of osimertinib-resistant EGFR^LR^ GEMMs. (**a**) Representative micro-CT-based 3D reconstructions showing lung tissue (green), tumor lesions (red), and trachea (yellow) at baseline (week 0, naïve stage), during response to osimertinib (week 4), and after acquisition of resistance at the end of treatment (week 42). White arrows indicate lesions that regress completely and do not re-grow, whereas yellow arrows mark lesions that become detectable during the resistant phase. (**b**) Tumor volume growth kinetics of individual lesions showing initial regression followed by tumor re-growth upon acquired resistance. Each curve indicates one representative lesion per mouse (*n* = 3) that remained measurable throughout treatment. Red arrows indicate naïve, responding (OsiS), and resistant-stage (OsiR) time points used for longitudinal lesion tracking.

In both EGFR^D19^ and EGFR^LR^ models, 3D representative reconstructions revealed comparable patterns of initial tumor regression followed by tumor re-growth (Figure 2a, Figure 3a and Supplementary Videos S1a–c, S2a–c). At baseline (naïve stage), multiple discrete lesions were detectable throughout the lungs.

Following treatment initiation, most lesions exhibited rapid volumetric regression within the first four weeks. A subset of lesions regressed completely and remained undetectable during prolonged therapy (Figure 2a, Figure 3a and Supplementary Videos S1a–c, S2a–c), suggesting durable suppression of disease to below the detection limit of micro-CT. Conversely, additional lesions became detectable at later time points (Figure 2a, Figure 3a and Supplementary Videos S1a–c, S2a–c), indicating heterogeneous lesion behavior during prolonged therapy.

For quantitative assessment, one or two lesions per mouse (*n* = 3 mice) that remained measurable and could be confidently localized throughout treatment was longitudinally tracked. In the EGFR^D19^ GEMMs, lesions underwent ~75–80% volume reduction by week 4, followed by stabilization until ~week 25–28, when tumor re-growth marked the onset of acquired resistance (Figure 2b).

Although resistance emerged around weeks 25–28, treatment was maintained beyond this time point to allow further expansion of resistant lesions, ensuring sufficient tumor volume at sacrifice.

In EGFR^LR^ GEMMs, initial regression was ~80–90% by week 4, with tumor volumes remaining largely stable over an extended period (weeks 4–38). During this phase, some lesions remained largely stable or showed modest decreases, while others became nearly undetectable, reflecting sustained tumor control under continuous therapy. Measurable re-growth began after week 38, and treatment continued until week 42 to allow resistant tumors to reach sufficient volume (Figure 3b).

For one of the three EGFR^LR^ mice, quantification was interrupted toward the end of treatment because the initially tracked lesion was no longer clearly detectable. Nonetheless, new dense regions and signs of localized inflammation emerging between weeks 38–42, particularly in the left lung lobe, may correspond to newly detectable lesions associated with resistant-stage disease (Supplementary Figure S3), supporting the development of resistance. For this mouse, longitudinal volumetric data are included up to and including week 38, the last time point at which the lesion was measurable. This animal is not included in quantitative comparisons at week 42.

Similar response kinetics were observed across the three animals within each genotype, supporting the consistency of these imaging observations within the exploratory cohort.

Together, EGFR^D19^ and EGFR^LR^ tumors displayed broadly similar response patterns, characterized by robust initial sensitivity to osimertinib and durable regression, although resistance emerged later in EGFR^LR^ tumors, indicating subtle allele-specific differences in resistance timing.

Overall, combined 3D reconstructions and longitudinal volumetric tracking enabled lesion-level assessment of treatment response and resistance timing in vivo.

### 2.3. Heterogeneous Response Patterns in EGFR^LT^ Tumors

**Figure 4:**
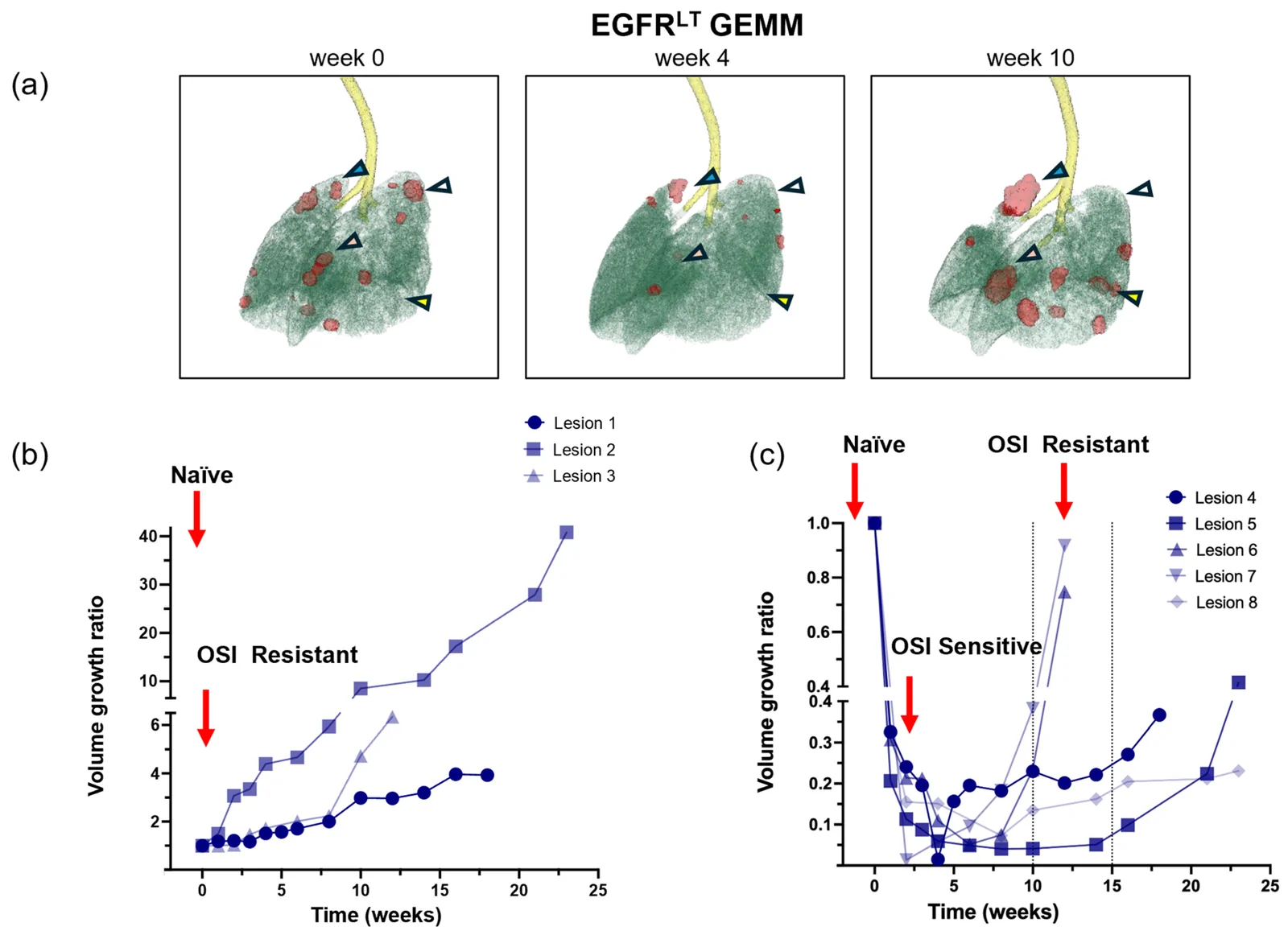
Generation of osimertinib-resistant EGFR^LT^ GEMMs. (**a**) Representative micro-CT-based 3D reconstructions showing lung tissue (green), tumor lesions (red), and trachea (yellow) at baseline (week 0, naïve stage), during response to osimertinib (week 4), and after acquisition of resistance (week 10). White arrows indicate lesions that respond to treatment and do not re-grow, whereas yellow arrows represent lesions that become detectable during the resistant phase. Blue arrows indicate lesions exhibiting continuous growth during therapy without measurable regression. Light orange arrows indicate lesions that show an initial response to treatment followed by re-growth during the resistant phase. (**b**,**c**) Tumor volume growth kinetics of individual lesions showing either continuous growth (**b**) or initial regression followed by tumor re-growth upon acquired resistance (**c**). Each curve indicates one defined lesion per mouse (*n* = 3 mice; *n* = 3 lesions in (**b**); and *n* = 3 mice; *n* = 5 lesions in (**c**)). Red arrows indicate naïve, responding (OsiS), and resistant-stage (OsiR) measurements.

By contrast, the EGFR^LT^ GEMMs revealed markedly heterogeneous patterns of response and resistance (Figure 4a and Supplementary Video S4a–c). At baseline (week 0), multiple discrete lesions were detected throughout the lungs. Following four weeks of osimertinib treatment, some lesions exhibited rapid volumetric regression (~70–80%) (Figure 4b and Supplementary Video S4a–c), whereas other lesions displayed limited or no regression, with early re-growth emerging between weeks 10–15 (Figure 4b,c and Supplementary Video S4a–c), substantially earlier than in EGFR^D19^ and EGFR^LR^ models.

A subset of lesions regressed completely and remained undetectable during prolonged therapy, suggesting durable suppression or loss of detectable disease within these lesions. In parallel, distinct lesions exhibited persistent growth during therapy without measurable regression (blue arrows, Figure 4a). New lesions became detectable at later time points, highlighting pronounced spatial heterogeneity in lesion behavior within individual lungs.

For quantitative assessment (Figure 4b,c), at least two lesions per mouse (*n* = 3) that remained measurable and could be confidently localized throughout treatment were longitudinally tracked, allowing representation of the heterogeneous lesion behaviors observed in the EGFR^LT^ model. This analysis identified distinct response patterns, including lesions exhibiting continuous growth through treatment (Figure 4b), and lesions showing initial regression followed by later re-growth (Figure 4c).

Collectively, EGFR^LT^ GEMMs exhibited highly heterogeneous response patterns, characterized by the coexistence of lesions showing continuous growth during therapy and initially responsive lesions that subsequently relapsed. These findings highlight the value of lesion-level monitoring for capturing spatial heterogeneity in therapeutic responses within individual lungs.

## 3. Discussion

Understanding how resistance to EGFR-targeted therapies emerges remains a central challenge in EGFR-mutant lung cancer. Third-generation EGFR inhibitors such as osimertinib have substantially improved outcomes in EGFR-mutant NSCLC, yet resistance inevitably develops. Therapeutic escape is associated with heterogeneous and often co-occurring mechanisms that vary across time, anatomical sites, and tumor cell populations [9,10,41]. Importantly, longitudinal imaging in this study revealed marked lesion-level heterogeneity in treatment response and resistance timing in vivo.

In this study, we established a longitudinal in vivo platform to investigate osimertinib resistance using GEMMs harboring clinically relevant EGFR mutations (EGFR^D19^, EGFR^LR^, and EGFR^LT^). By integrating high-resolution micro-CT imaging with lesion-specific volumetric tracking, we monitored tumor initiation, osimertinib response, and resistance timing in GEMMs carrying distinct EGFR genotypes. This approach enabled within-animal assessment of tumor behavior at both whole-lung and lesion-level resolution under sustained therapeutic pressure.

Our data indicate that EGFR mutational context is associated with differences in tumor growth kinetics and response to osimertinib. EGFR^LT^ tumors exhibited the most rapid onset of resistance, whereas EGFR^D19^ and EGFR^LR^ tumors showed slower growth and prolonged therapeutic control. These findings suggest that EGFR alleles are associated with distinct patterns of therapeutic response and resistance timing, consistent with previously reported mutation-dependent clinical outcomes and differential responses to EGFR-targeted therapies in patients [8,16,17,38,41].

Lesion-level analyses revealed marked intratumoral heterogeneity, particularly in EGFR^LT^ tumors, where lesions exhibiting continuous growth during therapy coexisted with lesions that initially responded but subsequently relapsed. In contrast, EGFR^D19^ and EGFR^LR^ tumors displayed more homogeneous response patterns, characterized by robust initial regression followed by delayed re-growth. Notably, across all EGFR genotypes, a subset of lesions underwent complete and durable regression, highlighting substantial spatial heterogeneity in lesion behavior within the same lung. These findings are consistent with current models of therapeutic resistance involving pre-existing resistant cell populations, drug-tolerant persister states, and therapy-induced adaptive processes [14,20,21,22,42,43,44,45]. However, the imaging data presented here do not resolve the cellular or clonal origin of individual lesions and therefore cannot distinguish among these potential mechanisms.

Our results also suggest an association between tumor growth rate and duration of response. Tumors with faster growth exhibited earlier resistance emergence, whereas slower-growing tumors displayed more prolonged sensitivity to osimertinib. Although this association requires validation in larger cohorts, it supports the value of longitudinal imaging for capturing genotype-associated differences in tumor behavior over time.

Importantly, conventional clinical readouts such as bulk imaging or circulating tumor DNA may obscure spatially distinct patterns of response occurring within the same lung. By contrast, longitudinal imaging enables direct visualization of treatment responses at the level of individual lesions. In this setting, lesion-level tracking provides information that is not captured by aggregate tumor burden alone, including the coexistence of regressing, persistent, re-growing, and newly detectable lesions within the same animal.

By enabling spatially-resolved monitoring during therapy, GEMMs combined with longitudinal imaging may serve as useful preclinical models to study response and resistance under clinically relevant selective pressures. Importantly, GEMMs preserve an intact immunocompetent tumor microenvironment, thereby maintaining continuous tumor-immune-stroma interactions during tumor progression. Previous single-cell analyses of EGFR-driven lung cancer models have highlighted the complexity of this tumor microenvironment [7]. Within this context, resistance develops within a physiologically relevant ecosystem rather than in tumor-intrinsic isolation.

By highlighting mutation-specific growth kinetics and spatial heterogeneity, this study underscores the importance of modeling diverse EGFR mutational contexts. The combination of EGFR-mutant GEMMs with longitudinal micro-CT imaging provides an experimental platform to monitor lesion-level response patterns and resistance timing during therapy. Finally, this imaging approach is readily adaptable to additional therapeutic contexts, including combination and sequential treatment strategies, and may help evaluate how different treatment regimens may shape response patterns over time.

Some limitations of the present study should be acknowledged. First, this work represents an exploratory proof-of-concept investigation with *n* = 3 mice per EGFR genotype. While the consistency of the observed response patterns supports the biological relevance of the findings, the limited cohort size constrains statistical power and generalizability. Validation in larger, independently-generated cohorts will be required to confirm the differences in tumor latency, response kinetics, and resistance timing reported here. Second, lesion classification was based exclusively on longitudinal imaging-derived volumetric measurements. Finally, although longitudinal lesion tracking reveals heterogeneous response patterns during therapy, imaging alone cannot determine the biological origin of lesions that subsequently re-grow. Such lesions may reflect residual disease persisting below the micro-CT detection threshold, late-emerging resistant lesions, or dissemination of resistant cells from other lesions within the same lung. Distinguishing among these possibilities will require dedicated histological and spatially-resolved molecular analyses beyond the scope of the present study.

In summary, longitudinal micro-CT imaging at single-lesion resolution revealed spatially heterogeneous, allele-associated patterns of tumor response and resistance timing during continuous osimertinib treatment in EGFR-mutant GEMMs.

## 4. Material and Methods

### 4.1. Establishment of GEMMs Colonies and Generation of OsiR GEMMs Models

Mice were housed in the CNR Animal Facility in Pisa under standard conditions, with food and water provided ad libitum and environmental enrichment provided.

Bitransgenic mice were generated by crossing TetO-regulated human EGFR mutant lines (EGFR^LT^, EGFR^D19^, and EGFR^LR^) with mice expressing reverse tetracycline transactivator under the Clara Cell Secretory Protein promoter (CCSP-rtTA) [46,47,48]. In this system, mutant EGFR expression is restricted to pulmonary epithelial cells and induced upon doxycycline administration, as previously described [7,49,50,51].

To initiate transgene expression and tumorigenesis, 8–10-week-old mice were fed a doxycycline-impregnated diet continuously. Lung tumor development was monitored by high-resolution micro-CT imaging performed every two weeks, and baseline tumor burden was established prior to treatment initiation.

Once tumors were detectable by imaging, mice were treated with osimertinib (10 mg/kg) administered by oral gavage twice weekly until acquired resistance was induced. Three mice per EGFR genotype were included in the study and monitored longitudinally over several months until resistance emergence. Treatment continued until evidence of tumor re-growth, consistent with the emergence of osimertinib resistance, was observed. Tumor burden was quantified by serial micro-CT imaging, initially weekly and then every two weeks, enabling lesion-specific longitudinal tracking. Lesions were tracked as imaging-defined anatomical entities across serial imaging time points and classified according to their longitudinal imaging behavior.

No a priori sample size calculation was performed. Sample size was determined based on prior experience with similar GEMM models.

Randomization was not formally applied, as experimental groups were defined by genetically engineered EGFR alleles, representing biologically distinct conditions. However, treatment was initiated when tumors reached comparable baseline sizes across animals, ensuring a balanced distribution of tumor burden between groups. Within each genotype, animals were enrolled consecutively and treated according to a consistent experimental schedule.

All experiments were performed in compliance with ethical regulations and approved by the Italian Ministry of Health (488/2022-PR). Animal health and welfare were monitored throughout the study. No unexpected adverse events were observed, and all procedures were conducted in accordance with approved ethical guidelines.

### 4.2. High Resolution Micro-CT Imaging

All micro-CT scans were performed with the IRIS scanner (Inviscan, Strasbourg, France). Prior to imaging, mice were pre-anaesthetized with isoflurane in an induction chamber and subsequently positioned on the imaging bed, where anesthesia was maintained using a continuous mixture of isoflurane and oxygen delivered via a nose mask at every imaging session, ensuring a reproducible and stable breathing rhythm across all time points. This minimized respiratory motion artefacts and supported consistent anatomical positioning between sessions, facilitating reliable longitudinal lesion tracking.

Scanning parameters were as follows: 65 kVp, 1 mA, 1280 projections over 360°, 70 ms of exposure time per projection, and a total scan time of 90 s. Images were corrected for beam hardening artifacts and reconstructed using cone-beam filtered backprojection (FBP) onto a 3D matrix of 600 × 600 × 1458 isotropic voxels, with a voxel size of 77 μm. Reconstructed datasets were exported in DICOM format for archiving and quantitative image analysis. Given the stable depth of sedation and regular free breathing pattern, respiratory gating was not required to obtain high-quality reconstructions of pulmonary lesions. In addition, the strong intrinsic contrast between solid tumor lesions and surrounding lung parenchyma allowed imaging without the use of exogenous contrast agents.

### 4.3. Tumor Lesion Quantification

Tumor volumes were quantified from DICOM micro-CT images using 3D Slicer (v5.8.0). For each mouse, one or two lesions that could be confidently localized across serial imaging time points were selected for longitudinal tracking. Additional lesions were reviewed for lesion-fate classification analyses whenever anatomical correspondence across serial imaging time points could be established with confidence. A dedicated segment was created for each lesion. Segmentation was performed using the Sphere Brush tool under masking conditions, applying the “Editable intensity range” option to restrict editing to predefined lesion intensity thresholds. Lesions were manually refined on a slice-by-slice basis to ensure accurate volumetric delineation and were systematically reviewed in both coronal and axial planes to confirm correct anatomical localization. Lesion inclusion criteria were as follows: (i) lesions were clearly identifiable at the naïve (baseline) imaging time point; (ii) anatomical correspondence could be confirmed across serial imaging sessions; and (iii) lesions could be delineated from surrounding tissue with sufficient confidence to allow reliable volumetric quantification. Lesion identity was established using anatomical correspondence across serial micro-CT datasets and confirmed through multi-planar review in axial and coronal views. Exclusion criteria included any imaging time points at which lesion identity or localization could not be assigned unambiguously, for example because of surrounding inflammation, below-threshold lesion size, or motion-related image blur. Such time points were excluded from quantitative analyses to minimize potential misclassification of lesion behavior over time.

Volumetric measurements were extracted using the Segment Statistics module, with identical segmentation thresholds applied consistently across all time points. The primary endpoint was labelmap (LM) volume (mm^3^). Lesion volumes were recorded at each time point until the development of resistance. Tumor growth volume dynamics were calculated relative to baseline (week 0, naïve tumor) and plotted for each EGFR mutant group. Blinding was not formally implemented during outcome assessment. However, lesion identification and volumetric quantification were based on standardized and objective imaging criteria, reducing potential operator-dependent bias.

### 4.4. Three-Dimensional Lung Reconstruction

Three-dimensional reconstruction and segmentation were performed using 3D Slicer (v5.8.0). For each EGFR-mutant GEMM group (EGFR^LT^, EGFR^D19^, and EGFR^LR^), one representative mouse was selected, and representative treatment time points were analyzed: pre-treatment (week 0), during response to osimertinib (OsiS, week 4), and at the final treatment time point.

DICOM micro-CT images were analyzed using the Segment Editor module. For lung and trachea segmentation, a new segment was created, and the Threshold tool was applied using an intensity range of −1024 to −300 Hounsfield Units (HU). Selected regions were verified in axial and coronal planes to ensure accurate inclusion of both lung lobes and trachea. Segmentation refinement was performed using the “Grow from Seeds” tool to optimize boundary delineation and exclude surrounding soft tissues.

For tumor lesion reconstruction, a separate segment was generated. Thresholding was applied using an intensity range of −200 to +3900 HU to identify tumor regions. Manual refinement was performed in axial, coronal, and sagittal planes when necessary to ensure accurate lesion contouring. Closed surface representation was used to generate 3D volumetric reconstructions. For visualization, lung opacity was set to 0.6, lesion opacity to 1.0, and background structures to 0.25. All segmentation parameters were kept constant across samples and time points to ensure reproducibility and comparability. Representative lesions shown in 2D axial and coronal reconstructions were selected based on their ability to be consistently localized across imaging sessions using the criteria described above (Supplementary Figure S5a–c).

### 4.5. Statistics Analysis and Graphs

Graphs were generated using GraphPad Prism 10.4.1 (RRID:SCR_002798). Given the exploratory nature of the study and the limited sample size (*n* = 3 mice per EGFR genotype), analyses were descriptive and were not intended for definitive genotype-level inference. Longitudinal lesion measurements are therefore presented descriptively, focusing on temporal trends and imaging-defined lesion behavior over time.

## Data Availability

The data underlying this study, including raw imaging data, lesion annotations, and analysis files, are available from the corresponding author upon reasonable request.

## Supplementary Materials

The following supporting information can be downloaded at: https://www.mdpi.com/article/10.3390/ijms27177727/s1.
