# Supplementary material for: Longitudinal In Vivo Imaging at Single-Lesion Resolution Identifies Allele-Associated Response and Resistance Dynamics in EGFR-Mutant Lung Cancer
Source: Int J Mol Sci. 2026 Aug 28;27(17):7727. doi: 10.3390/ijms27177727 (PMC13566647; doi:10.3390/ijms27177727)
Supplement: Supplementary file 1 [file ijms-27-07727-s001.zip › ijms-4369226-supplementary.pdf]

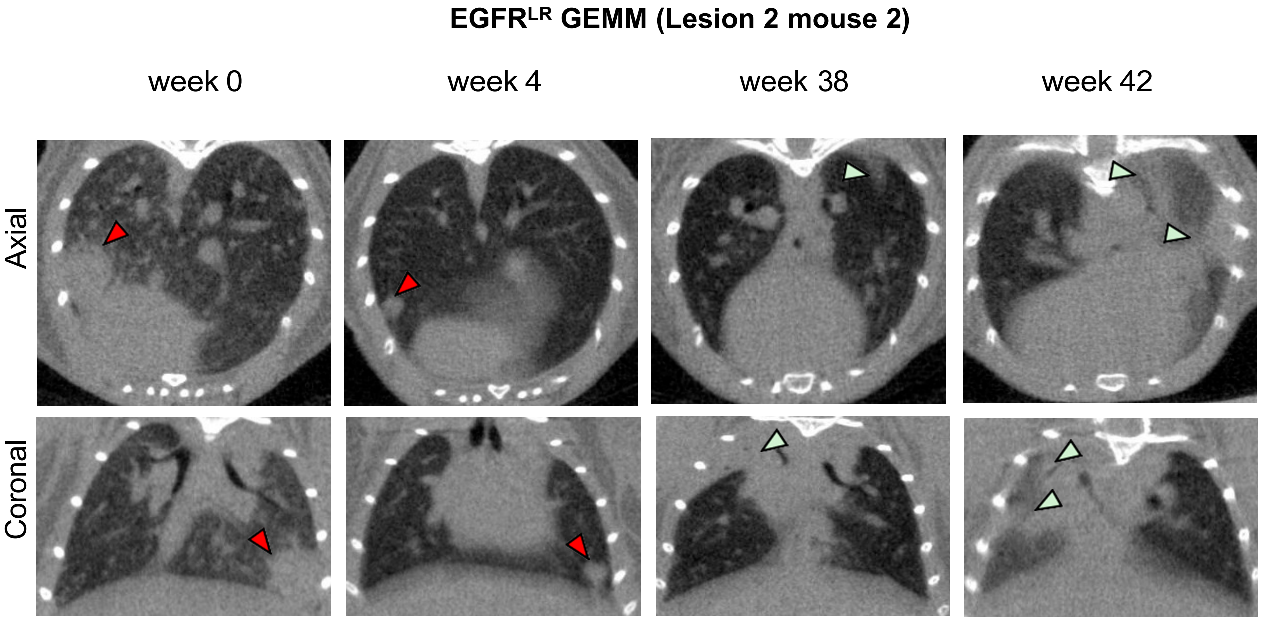


**Supplementary Fig. S3. Representative 2D micro-CT images of the OsiR EGFR$^{LR}$ model.** Representative 2D micro-CT images in axial (top) and coronal (bottom) views from one of $n$=3 mice in the OsiR EGFR$^{LR}$ GEMM group. Images are shown at baseline (week 0, treatment-naïve stage), during response to osimertinib (week 4), and after acquisition of resistance at the end of treatment (weeks 38-42). Red arrowheads indicate quantified lesions that initially responded to treatment but subsequently were undetectable. Green arrowheads indicate areas of inflammation and regions potentially corresponding to emerging resistant lesions for which lesion identity and anatomical correspondence could not be assigned with sufficient confidence for reliable longitudinal quantification, as they are partially obscured by inflammation. This figure illustrates the temporal dynamics of lesion regression, the emergence of resistant lesions, and the challenges associated with lesion tracking and quantification in the presence of inflammation, complementing the growth curve analysis.

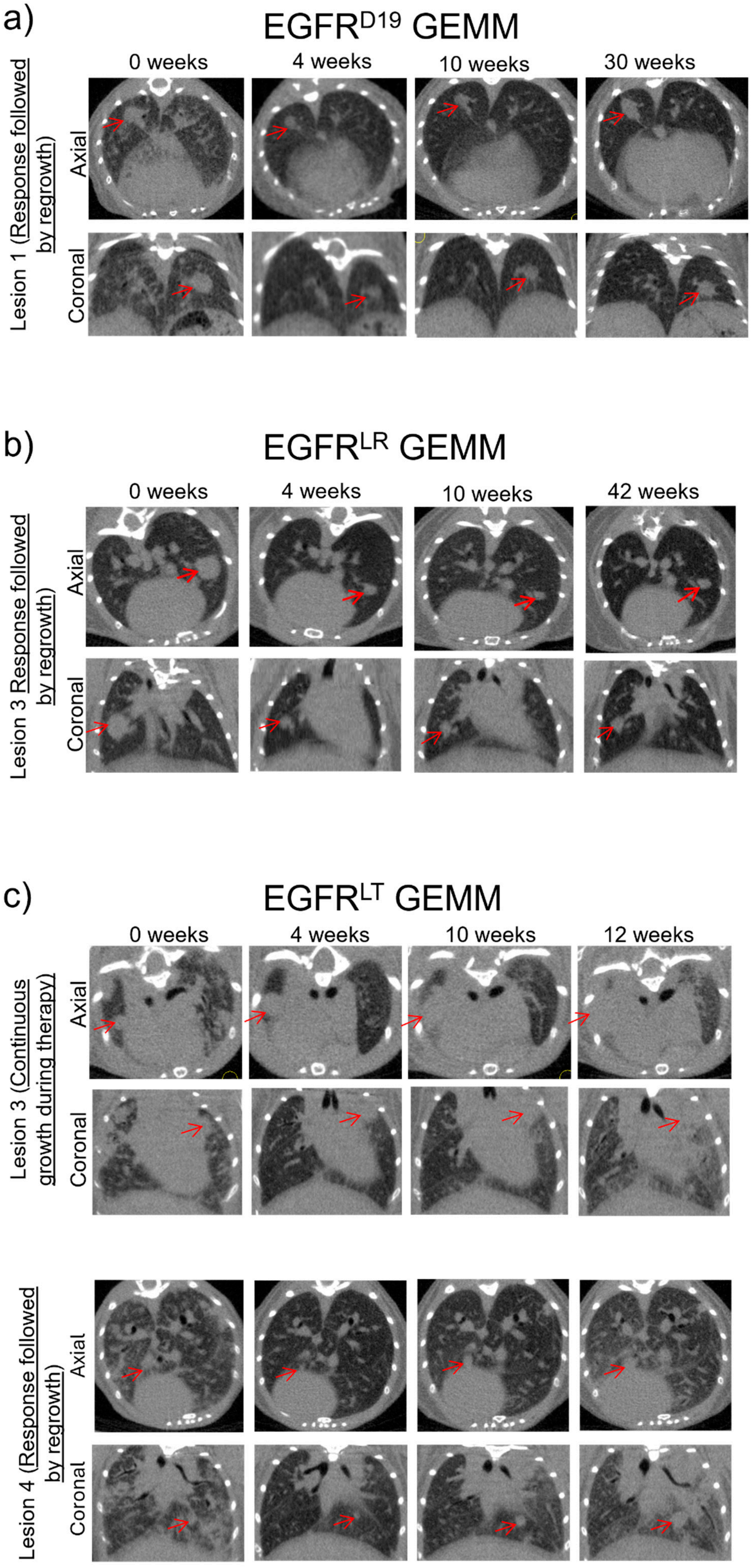
a)
EGFR$^{D19}$ GEMM
0 weeks
4 weeks
10 weeks
30 weeks
Lesion 1 (Response followed by regrowth)
Axial
Coronal
b)
EGFR$^{LR}$ GEMM
0 weeks
4 weeks
10 weeks
42 weeks
Lesion 3 Response followed by regrowth)
Axial
Coronal
c)
EGFR$^{LT}$ GEMM
0 weeks
4 weeks
10 weeks
12 weeks
Lesion 3 (Continuous growth during therapy)
Axial
Coronal
Lesion 4 (Response followed by regrowth)
Axial
Coronal

**Supplementary Fig. S5. Longitudinal per-lesion micro-CT imaging across EGFR-mutant GEMM genotypes**. Representative 2D micro-CT images in axial (top) and coronal (bottom) views of individual lesions longitudinally tracked in $EGFR^{D19}$, $EGFR^{LR}$, and $EGFR^{LT}$ GEMMs. Red arrows indicate the lesion tracked at each timepoint. (a) Lesion 1 from an $EGFR^{D19}$ GEMM mouse, imaged at weeks 0, 4, 10, and 30, (b) Lesion 3 from an $EGFR^{LR}$ GEMM mouse, imaged at weeks 0, 4, 10, and 42. (c) Two lesions (3 and 4) from the same $EGFR^{LT}$ GEMM mouse. Lesion 3 (top), imaged at weeks 0, 4, 10, and 12, shows continuous growth throughout therapy, whereas Lesion 4 (bottom), imaged over the same interval, initially shows response to treatment followed by regrowth.
